# Iron-Bound Lipocalin-2 Protects Renal Cell Carcinoma from Ferroptosis

**DOI:** 10.3390/metabo11050329

**Published:** 2021-05-19

**Authors:** Julia K. Meier, Matthias Schnetz, Susanne Beck, Tobias Schmid, Monica Dominguez, Sanela Kalinovic, Andreas Daiber, Bernhard Brüne, Michaela Jung

**Affiliations:** 1 Faculty of Medicine, Institute of Biochemistry I, Goethe-University Frankfurt, 60590 Frankfurt am Main, Germany; meier@biochem.uni-frankfurt.de (J.K.M.); matthias.schnetz@t-online.de (M.S.); t.schmid@biochem.uni-frankfurt.de (T.S.); dominguez@biochem.uni-frankfurt.de (M.D.); b.bruene@biochem.uni-frankfurt.de (B.B.); 2 Institute of Pathology, University Hospital Heidelberg, 69120 Heidelberg, Germany; Susanne.Beck@med.uni-heidelberg.de; 3 Department of Cardiology 1, Molecular Cardiology, Medical Center of the Johannes Gutenberg University, 55131 Mainz, Germany; sanelakalinovic@gmail.com (S.K.); Daiber@uni-mainz.de (A.D.); 4 Fraunhofer Institute for Translational Medicine and Pharmacology, 60596 Frankfurt am Main, Germany; 5 German Cancer Consortium (DKTK), Partner Site Frankfurt, 60590 Frankfurt am Main, Germany; 6 Frankfurt Cancer Institute, Goethe-University Frankfurt, 60596 Frankfurt am Main, Germany

**Keywords:** lipocalin-2, iron, ROS, *SLC7A11*, *Nrf2*, ISR, p-eIF2α, erastin, ferroptosis

## Abstract

While the importance of the iron-load of lipocalin-2 (Lcn-2) in promoting tumor progression is widely appreciated, underlying molecular mechanisms largely remain elusive. Considering its role as an iron-transporter, we aimed at clarifying iron-loaded, holo-Lcn-2 (hLcn-2)-dependent signaling pathways in affecting renal cancer cell viability. Applying RNA sequencing analysis in renal CAKI1 tumor cells to explore highly upregulated molecular signatures in response to hLcn-2, we identified a cluster of genes (*SLC7A11, GCLM, GLS*), which are implicated in regulating ferroptosis. Indeed, hLcn-2-stimulated cells are protected from erastin-induced ferroptosis. We also noticed a rapid increase in reactive oxygen species (ROS) with subsequent activation of the antioxidant *Nrf2* pathway. However, knocking down *Nrf2* by siRNA was not sufficient to induce erastin-dependent ferroptotic cell death in hLcn-2-stimulated tumor cells. In contrast, preventing oxidative stress through *N*-acetyl-l-cysteine (NAC) supplementation was still able to induce erastin-dependent ferroptotic cell death in hLcn-2-stimulated tumor cells. Besides an oxidative stress response, we noticed activation of the integrated stress response (ISR), shown by enhanced phosphorylation of eIF-2α and induction of *ATF4* after hLcn-2 addition. *ATF4* knockdown as well as inhibition of the ISR sensitized hLcn-2-treated renal tumor cells to ferroptosis, thus linking the ISR to pro-tumor characteristics of hLcn-2. Our study provides mechanistic details to better understand tumor pro-survival pathways initiated by iron-loaded Lcn-2.

## 1. Introduction

One of the most recurrent urologic tumors is renal cell carcinoma (RCC). Due to its clinically silent development and progression, RCC represents a major, yet underestimated health problem, since the detection of the majority of RCC cases is by incidental radiologic discovery [1]. Resistance of RCC against chemo- and radiation-therapy further restricts therapeutic medical options [2]. Currently, no reliable diagnostic approach for early RCC detection and no effective method for recurrence surveillance or therapy response are available. It appears pivotal to identify suitable diagnostic and prognostic biomarkers, in order to enable detection of premetastatic tumors and to install efficient therapy approaches such as partial rather than radical nephrectomy. An unmet need also represents a more sophisticated in-depth understanding of underlying molecular disease mechanisms as well as crucial signaling pathways and/or molecules for targeted interference strategies.

With regard to drug resistance, a common approach in cancer therapy is the activation of regulated cell death. It is commonly appreciated that tumor cells not only re-wire their oxygen-consuming metabolic activity, which is regulated by activation of hypoxia-inducible factor 1 (HIF-1), but also enhance their resistance against oxidative stress and reactive oxygen species (ROS)-associated cellular damage [3]. Recently, a new form of regulated cell death named ferroptosis was described that links the availability of intracellular iron to the induction of cytotoxic lipid peroxides. Ferroptosis is both morphologically and biochemically different to other regulated cell death forms [4]. Renal cell carcinomas are particularly susceptible to ferroptotic cell death, since they are exceptionally dependent on glutathione synthesis to prevent lipid peroxidation and thus, to maintain cellular survival and viability [5]. Glutathione peroxidase 4 (GPX4) is known to protect cells from ferroptosis by converting glutathione (GSH) into oxidized glutathione (GSSG), thereby reducing cytotoxic lipid peroxides (L-OOH) to the corresponding non-toxic alcohols (L-OH) [6,7]. Upon inhibition of GPX4, intracellular iron in its Fe^2+^ form oxidizes lipids in a Fenton-like manner, which, in turn, causes ROS accumulation and cell death [8,9]. Along these lines, siRNA screening approaches indicated that blocking genes encoding either glutathione peroxidases GPX3 or GPX4 is particularly lethal to renal tumor cells [10].

The activity of GPX4 is closely linked to the Xc-system, acting as an antiporter for cystine and glutamate that is necessary for GSH synthesis [11]. Moreover, it was previously shown that inhibition of the Xc-system significantly lowers cancer cell resistance to conventional radio- and chemotherapy [12]. Since iron is required for the execution of ferroptosis due to its function in the accumulation of lipid peroxides, it is crucial to understand how iron turnover is controlled in tumor cells and how iron availability impacts tumor cell sensitivity to ferroptosis. Along these lines, previous studies noticed that the transferrin (Tf)-transferrin receptor 1 (TfR)-system is required for ferroptotic cell death [8], whereas inhibiting the master regulator of cellular iron homeostasis, iron-responsive element-binding protein 2 (IRP-2), enhances tumor cell resistance towards ferroptosis [11]. An additional important mechanism to control cellular sensitivity to ferroptosis is ferritinophagy, whereby ferritin is recognized by nuclear receptor coactivator 4 (NCOA4) to be recruited to autophagosomes and marked for lysosomal degradation [13,14,15]. Nonetheless, despite iron being considered a hallmark of ferroptotic cell death, mechanistic implications of iron other than promoting Fenton chemistry are still not fully understood. However, a recent report from Müller et al. nicely showed that not only common players in iron metabolism such as TfR should be considered in the tumor context. The authors showed that CD44 adopts the role of iron endocytosis, whereby the epigenetic plasticity of cancer cells is critically shaped and the expression of specific genes of the mesenchymal cell state is fostered [16].

It is widely accepted that cancer cells require high amounts of iron in order to sustain an enhanced metabolic turnover and to increase proliferation [17]. Tumor cells adopt an iron-retaining phenotype [18], thereby maintaining a delicate balance of metabolically required iron and cytotoxic effects of excessive iron. In particular, cancer stem cells were found to massively increase iron uptake via enhanced TfR expression and iron mobilization through increased ferritin levels. Most interestingly, cancer stem cells seem to show an iron-phenotypic molecular fingerprint that allows the adaptation of cancer stem cells to microenvironmental cues of the respective niche [19]. Moreover, interfering with proper iron storage such as blocking ferritin, was detrimental to cells addicted to high iron levels and represents a potent target for novel therapeutic avenues. In line, Mai et al. showed that targeting cancer stem cells by using a synthetic derivative of salinomycin significantly enhanced iron retention in lysosomes, whereby iron-dependent ROS formation fostered lysosomal membrane rupture, finally leading to iron-dependent ferroptotic cell death [20].

We and others have previously described that tumor-associated macrophages (TAM) represent a major source of iron for the growing tumor [21,22], releasing significant amounts of iron to the tumor microenvironment [23]. Moreover, we showed that TAM release iron, bound to the iron-transporting protein lipocalin-2, which, in turn, promoted tumor growth in an experimental mammary tumor model [21]. Enhanced levels of lipocalin-2 (Lcn-2) were previously also associated with reduced disease-free survival in breast cancer patients [24], while inhibition of Lcn-2 significantly reduced mammary tumor progression and metastasis [25,26,27]. Considering the role of Lcn-2 in RCC pathogenesis, previous studies identified increased amounts of Lcn-2 in both urine and serum of RCC patients [28,29], and the expression of Lcn-2 protein in tumor tissues positively correlated with histological grades in clear cell RCC (ccRCC) and papillary RCC (pRCC) patients [30]. Recently, we provided evidence that the iron-binding capacity of Lcn-2 defines its pro-tumor characteristics in ccRCC [31]. Iron-loaded Lcn-2 promoted RCC tumor growth and progression, whereas the iron-free form of Lcn-2 showed rather anti-tumoral activity, while mechanistic details on Lcn-2 signaling still remain obscure.

Here we explored how iron-loaded Lcn-2 drives renal tumor growth and progression and identified a crucial role of the Xc-system. hLcn-2 significantly enhances cellular resistance to ferroptosis, by inducing the antioxidant kelch-like ECH-associated protein 1 (Keap1)/nuclear factor erythroid 2-related factor 2 (*Nrf2*) pathway and causing an integrated stress response through p-eIF2α (phosphorylated eukaryotic translation initiation factor 2A) activation.

## 2. Results

### 2.1. Iron-Loaded Lcn-2 Induces Genes Referring to Glutathione Biosynthesis

**Figure 1:**
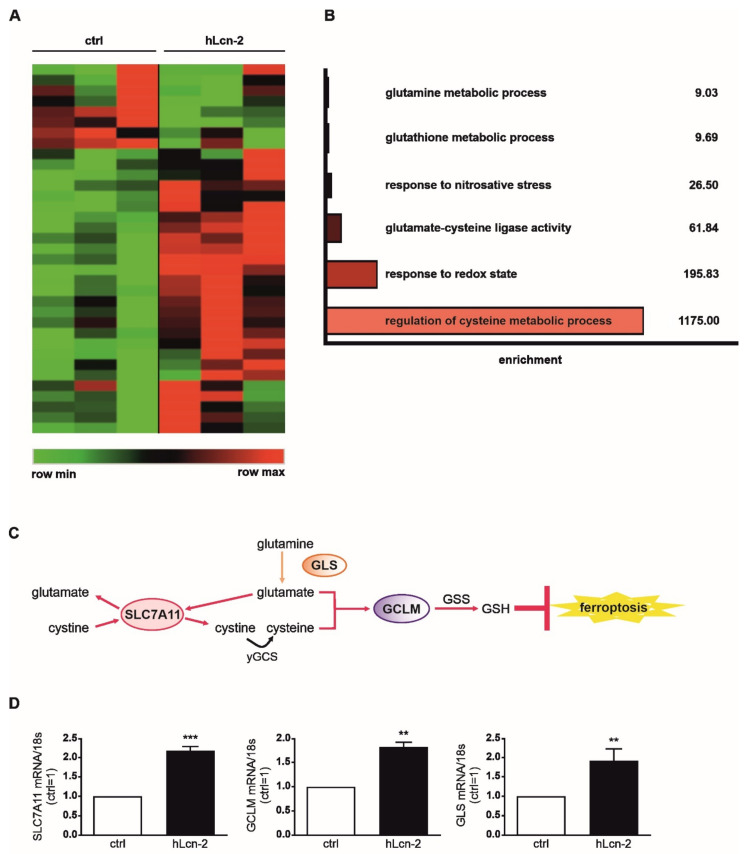
RNAseq analysis reveals a hLcn-2-induced cluster of genes involved in the regulation of ferroptosis. If not stated otherwise Lcn-2 treatments were with 5 µg/mL for 24 h in all experiments. (**A**) Heatmap of differentially expressed genes between iron-loaded, holo-Lcn-2 (hLcn-2) stimulated renal CAKI1 tumor cells and unstimulated control (ctrl) cells (*n* = 3). (**B**) GO-term analysis and graph of enriched GO-terms. Enrichment is defined as: (b/n)/(B/N) (N = total number of genes, B = total number of genes associated with a specific GO-term, *n* = number of genes in the top of the target set, b = number of genes in the intersection). (**C**) Schematic representation of the glutathione pathway with highlighted target genes *SLC7A11*, *GCLM*, and *GLS* in bold. (**D**) mRNA expression of *SLC7A11*, *GCLM*, and *GLS* in hLcn-2-stimulated cells in comparison to untreated cells normalized to the housekeeping gene *18S* (*n* = 4). Graphs are displayed as means ± SEM with ** *p* < 0.01, *** *p* < 0.001.

Since we previously determined that the iron-load of Lcn-2 plays a critical role for its pro-tumor functions in RCC [31], we now aimed to identify underlying molecular mechanisms. First, we performed RNAseq analysis of renal CAKI1 tumor cells stimulated with 5 µg/mL hLcn-2 for 24 h (Figure 1) and identified 35 mRNAs being differentially expressed (*p* ≤ 0.05) comparing controls and hLcn-2-treated samples (Figure 1A and Supplemental Figure S1A). Gene ontology (GO)-term analysis applying GOrilla showed enrichment of glutamine metabolic process, glutathione metabolic process, response to nitrosative stress, glutamate-cysteine ligase activity, response to the redox state, and regulation of cysteine metabolic process (Figure 1B). Of note, we identified *SLC7A11* (solute carrier family 7 member 11), *GCLM* (glutamate-cysteine ligase modifier subunit), and *GLS* (glutaminase), which cluster within the GO-terms, referring to glutathione biosynthesis and inhibiting ferroptotic cell death (Figure 1C). For validation, we analyzed their mRNA expression in renal CAKI1 (Figure 1D) and renal A498 cells (Supplemental Figure S1B) as well as mammary MCF-7 cells (Supplemental Figure S1C) and observed significantly increased mRNA expression in hLcn-2-stimulated cells compared to untreated controls. By comparing hLcn-2-effects in primary human tubular epithelial cells (TEC) to primary tumor cells (TTEC), we observed that all identified target genes were only induced in tumor cells, whereas healthy cells did not respond (Supplemental Figure S1D).

**Figure 2:**
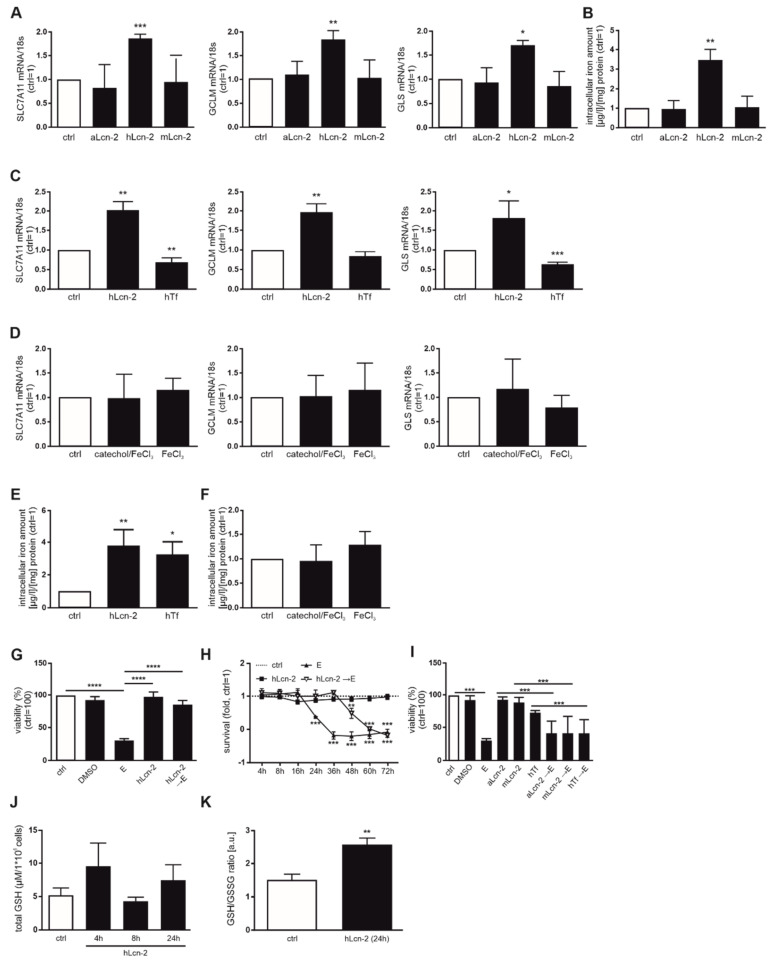
hLcn-2 delays erastin-induced ferroptosis. (**A**) mRNA expression of *SLC7A11*, *GCLM*, and *GLS* after stimulation with iron-free apo-Lcn-2 (aLcn-2), holo-Lcn-2 (hLcn-2), or the no iron-binding mutant Lcn-2 (mLcn-2) (*n* = 4). (**B**) Measurement of intracellular iron amount by AAS analysis after Lcn-2 stimulation (*n* = 4). (**C**,**D**) *SLC7A11*, *GCLM*, and *GLS* mRNA expression after (**C**) holo-Lcn-2 (hLcn-2) compared to holo-Transferrin (hTf) (*n* = 4), or (**D**) iron-loaded catechol (catechol/FeCl_3_) and FeCl_3_. mRNA expression was normalized to housekeeping gene *18S* expression (*n* = 4). (**E**,**F**) AAS measurements of CAKI1 cells stimulated with (**E**) hLcn-2 compared to hTf (*n* = 4) or (**F**) iron-loaded catechol (catechol/FeCl_3_) compared to FeCl_3_ alone (*n* = 4). (**G**,**H**) CAKI1 cells were pre-stimulated with hLcn-2 for 24 h, washed and incubated with erastin (10 µM) for additional 24 h. (**G**) Cell viability measured with CellTiter Blue assay and normalized to the unstimulated control. DMSO served as solvent control (*n* = 4). (**H**) Survival assay accomplished with xCELLigence RTCA real-time measurement. A DMSO-stimulated control served for normalization (*n* = 3). (**I**) Cell viability measured with CellTiter Blue assay and normalized to the unstimulated control. DMSO served as solvent control. CAKI1 cells were pre-stimulated with either aLcn-2, mLcn-2, or hTf for 24 h, washed and incubated with erastin for additional 24 h (*n* = 4). (**J**) Measurement of total GSH by HPLC (*n* = 4) as well as (**K**) the ratio of GSH to GSSG by a colorimetric assay in CAKI1 cells after stimulation with hLcn-2 for indicated timepoints (*n* = 4). Graphs are displayed as means ± SEM with * *p* < 0.05, ** *p* < 0.01, *** *p* < 0.001, **** *p* < 0.0001.

In order to verify whether *SLC7A11*, *GCLM*, and *GLS* are specifically induced by iron-loaded Lcn-2, we stimulated CAKI1 tumor cells with iron-free, apo-Lcn-2 (aLcn-2) and a mutant form (mLcn-2) deficient of its iron binding capacity compared to hLcn-2 (Figure 2A). There was no target gene response with either aLcn-2 or mLcn-2. As a control, the intracellular iron amount of CAKI1 was measured, indicating that only hLcn-2 increased the intracellular iron amounts (Figure 2B). To explore whether specifically iron, transported by hLcn-2, induces gene activation, we employed iron-loaded transferrin (hTf), FeCl_3_, or the Lcn-2-binding catechol/FeCl_3_-complex as controls. As seen in Figure 2C,D, none of these stimuli induced the target genes *SLC7A11*, *GCLM*, and *GLS*. As an additional control, we verified intracellular iron amounts in CAKI1 cells following hTf-, FeCl_3_-, or catechol/FeCl_3_-complex-stimulation. Only hTf increases intracellular iron levels comparable to hLcn-2 (Figure 2E), whereas no enhanced intracellular iron levels were observed for FeCl_3_ or the catechol/FeCl_3_-complex (Figure 2F). Thus, target gene expression is induced specifically upon stimulation with iron-loaded Lcn-2, whereas the stimulation with iron per se or iron-loaded Tf remained without effect.

### 2.2. hLcn-2 Protects Renal Tumor Cells against Erastin-Induced Ferroptotic Cell Death

Considering the association between *SLC7A11*, *GCLM* and *GLS* with ferroptosis, we hypothesized that hLcn-2 may protect renal tumor cells against this form of cell demise. To test this, we pre-stimulated CAKI1 tumor cells with hLcn-2 for 24 h before adding the ferroptosis-inducing agent erastin. 10 µM erastin, supplied for 24 h to CAKI1 cells, reduced viability by roughly 50% (Supplemental Figure S2A), while hLcn-2-pre-stimulation significantly protected against erastin-induced cell death (Figure 2G). These observations were validated applying xCELLigence real-time measurements of cellular survival over a period of 3 days. hLcn-2 pre-stimulation significantly delayed the ferroptotic cell death response towards erastin (for about 36 h) (Figure 2H). We also applied ferrostatin-1 (fer-1), an established ferroptosis inhibitor, as well as z-vad-fmk, a pan caspase inhibitor to block apoptosis, to specify ferroptotic cell death (Supplemental Figure S2B). Our results indicate that only fer-1 as an inhibitor of ferroptosis was able to restore cellular viability upon erastin-treatment, whereas z-vad-fmk as inhibitor of the apoptotic cell death pathway remained without effect upon erastin-stimulation. In order to prove specificity for hLcn-2, we pre-stimulated CAKI1 cells also with apo- and mutant Lcn-2 as well as hTf, before inducing ferroptotic cell death applying erastin. Results show that only hLcn-2, but neither aLcn-2 nor mLcn-2 or Tf protected cells from ferroptosis (Figure 2I).

Since inhibition of ferroptosis is linked to GSH biosynthesis, we analyzed total GSH in CAKI1 cells after 4, 8, and 24 h of hLcn-2 stimulation by high-performance liquid chromatography (HPLC). We observed slightly, but not significantly increased amounts of total GSH at 4 and 24 h after hLcn-2 stimulation (Figure 2J). A colorimetric GSH assay confirmed results in terms of total GSH (Supplemental Figure S2C). We further noticed lower amounts of GSSG (Supplemental Figure S2D) and increased levels of reduced GSH at 24 h after hLcn-2 stimulation (Supplemental Figure S2E), accounting for a higher GSH to GSSG ratio (Figure 2K), pointing to a protective effect of hLcn-2. Along these lines, we observed a higher expression of the key enzyme GPX4 (Supplemental Figure S2F).

### 2.3. hLcn-2 Induces a Rapid ROS-Response and Activates the Keap-1/Nrf2 Pathway

**Figure 3:**
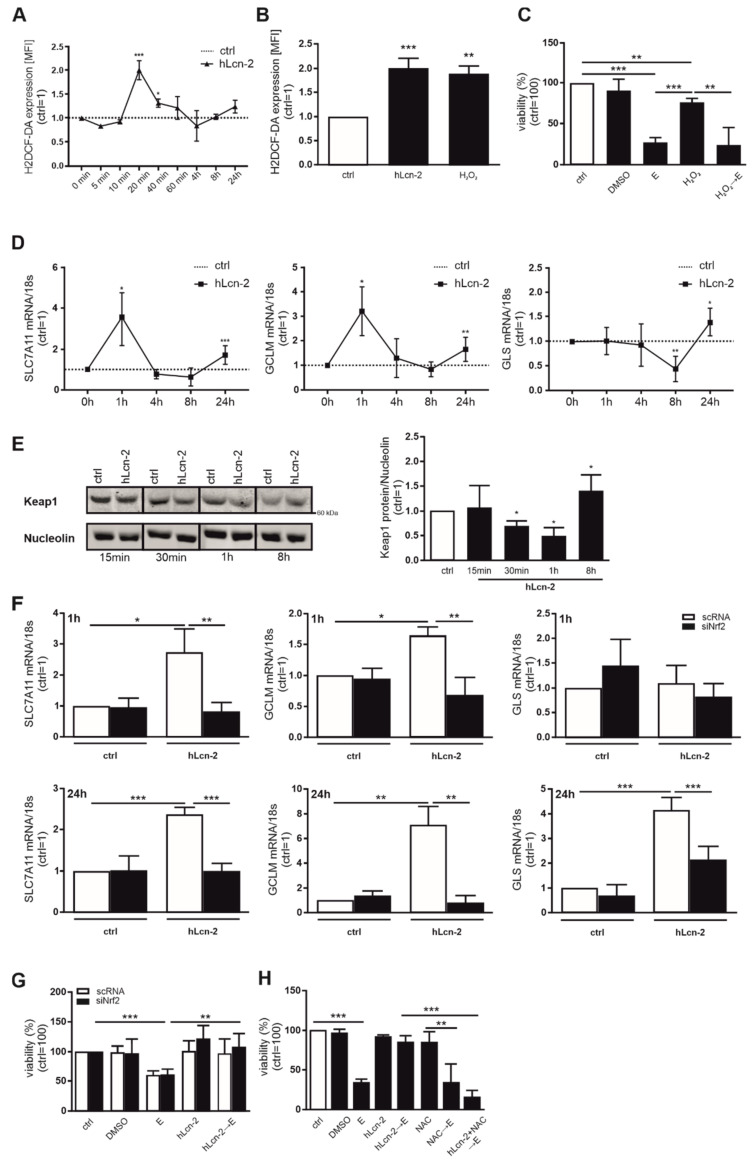
hLcn-2 fosters Keap1/*Nrf2* pathway by inducing ROS. (**A**,**B**) CAKI1 cells were stimulated with 5 µg/mL hLcn-2 for the times indicated. H2DCF-DA was applied to measure the appearance of ROS. Results were analyzed as mean fluorescence intensity (MFI) by flow cytometry and are expressed normalized to the untreated control for each time point, showing (**A**) ROS induction from 5 min to 24 h after hLcn-2 stimulation (*n* = 4) and (**B**) similar amounts of ROS following hLcn-2 stimulation or H_2_O_2_ addition for 20 min (*n* = 4). (**C**) Cell viability measured with CellTiter Blue assay, normalized to unstimulated controls. DMSO served as solvent control. CAKI1 cells were pre-stimulated with H_2_O_2_ (500 µM) for 24 h, washed and incubated with erastin (10 µM) for additional 24 h (*n* = 4). (**D**) mRNA expression of *SLC7A11*, *GCLM*, and *GLS* after hLcn-2 (5 µg/mL) for the indicated time points. mRNA expression was normalized to housekeeping gene *18S* expression (*n* = 4). (**E**) Western analysis of Keap1 protein degradation after the stimulation with 5 µg/mL hLcn-2 for the indicated time points. Nucleolin was analyzed as loading control. A representative picture from 4 independent experiments is given along with the densitometrical analysis (*n* = 4). (**F**,**G**) CAKI1 cells were treated with either a siRNA against *Nrf2* (siNrf2) or a scrambled control RNA (scRNA) before (**F**) stimulating the cells with 5 µg/mL hLcn-2 for 1 h (upper panels) or 24 h (lower panels) (*n* = 4). mRNA expression of *SLC7A11*, *GCLM*, and *GLS* was analyzed relative to the housekeeping gene *18S* expression and results are represented normalized to scRNA-treated control cells (*n* = 4). (**G**) Cell viability measured with CellTiter Blue assay, normalized against the unstimulated control. DMSO served as solvent control. scRNA- or siNrf2-CAKI1 cells were pre-stimulated with 5 µg/mL hLcn-2 for 24 h, washed, and incubated with erastin (10 µM) for additional 24 h (*n* = 4). (**H**) Cell viability assay normalized to the unstimulated control. DMSO served as solvent control. CAKI1 cells were co-stimulated with 5 µg/mL hLcn-2 and *N*-acetyl-l-cysteine (NAC; 10 mM) for 24 h, washed, and incubated with erastin (10 µM) for additional 24 h (*n* = 4). Graphs are displayed as means ± SEM with * *p* < 0.05, ** *p* < 0.01, *** *p* < 0.001.

Considering that hLcn-2 transports iron into renal tumor cells, we explored potential consequences. Measuring ROS, using H2DCF-HA, we noticed a peak after 20 min, with declining levels afterwards (Figure 3A). The extent to which hLcn-2-promoted ROS was like that provoked by 500 µM H_2_O_2_ (Figure 3B). However, ROS alone was not sufficient to protect from erastin-induced cell death (Figure 3C). Analyzing the time course of mRNA induction of target genes, we noticed induction of *SLC7A11* and *GCLM* already after 1 h and a much lower response for all three targets at 24 h, whereas *GLS* only peaked at 24 h (Figure 3D). This allowed to speculate about a rapid, ROS-dependent Keap1/*Nrf2* pathway activation that might be involved in subsequent target gene expression. Analyzing degradation of Keap1 by Western blot analysis, we found a rapid degradation after 30 min to 1 h of hLcn-2 stimulation and normalization of Keap1 levels after 8 h (Figure 3E). To test whether the Keap1-regulated transcription factor *Nrf2* is involved in controlling *SLC7A11*, *GCLM*, and *GLS* expression, we performed a knockdown of *Nrf2* (siNrf2). This significantly reduced hLcn-2-induced *Nrf2* expression (Supplemental Figure S3A) and suppressed its target genes NAD(P)H quinone dehydrogenase 1 (NQO1) and heme oxygenase 1 (HO-1) (Supplemental Figure S3B) compared to scrambled control (scRNA) transfected cells. In *Nrf2*-depleted cells, expression of *SLC7A11*, *GCLM*, and *GLS* were significantly reduced at 1 h (except for *GLS*, which was not regulated at the 1 h timepoint) and 24 h after hLcn-2 stimulation (Figure 3F). However, knocking down *Nrf2* did not influence the ability of hLcn-2 to protect against erastin-induced ferroptosis (Figure 3G). Analyzing the viability of tumor cells either transfected with a scRNA or siRNA against *Nrf2*, revealed that hLcn-2 protects against cell death in the presence or absence of *Nrf2*. Interestingly, when co-stimulating tumor cells with hLcn-2 and the antioxidant NAC prior to adding erastin, protection was lost (Figure 3H).

Thus, while the Keap1/*Nrf2* pathway is involved in regulating hLcn-2 target gene expression, it does not directly convey protection. Nevertheless, since NAC overcame the protective role of Lcn-2, a ROS-dependent mechanism seems likely.

### 2.4. hLcn-2 Stimulation Induces an Integrated Stress Response

**Figure 4:**
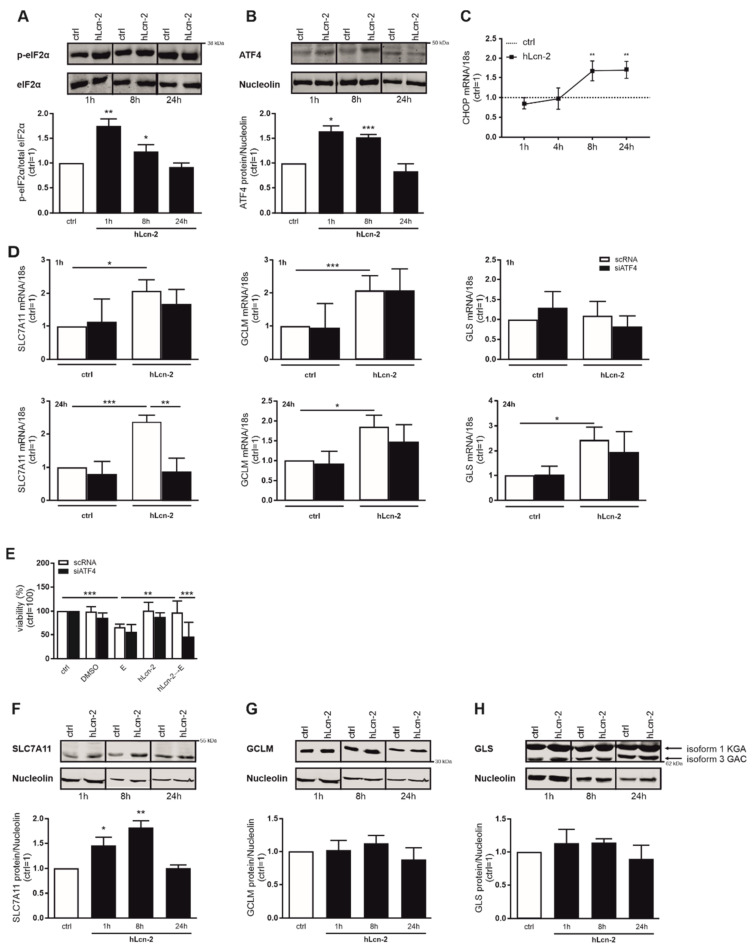
hLcn-2 induces the phosphorylation of eIF2α with subsequent activation of ATF4 and *SLC7A11*. (**A**,**B**) Western analysis of (**A**) p-eIF2α (*n* = 3) and (**B**) ATF4 protein expression after the stimulation with 5 µg/mL hLcn-2 for the indicated time points. Either total eIF2α or nucleolin was analyzed as a loading control. A representative picture (upper panel) from 4 independent experiments is given along with the densitometrical analysis (lower panel) (*n* = 3). (**C**) mRNA expression of *CHOP* after hLcn-2 (5 µg/mL) for the indicated time points. mRNA expression was normalized to housekeeping gene *18S* expression (*n* = 4). (**D**,**E**) CAKI1 cells were treated with either a siRNA to knockdown *ATF4* (siATF4) or a scrambled control RNA (scRNA) before stimulation with 5 µg/mL hLcn-2 for (**D**) 1 h (upper panel) or 24 h (lower panel). mRNA expression of *SLC7A11*, *GCLM*, and *GLS* was analyzed relative to the housekeeping gene *18S* expression and results are represented normalized to scRNA-treated control cells (*n* = 4). (**E**) Cell viability measured with CellTiter Blue assay, normalized to unstimulated controls. DMSO served as solvent control. scRNA- or siATF4-CAKI1 cells were pre-stimulated with 5 µg/mL hLcn-2 for 24 h, washed, and incubated with erastin (10 µM) for additional 24 h (*n* = 4). (**F**–**H**) Western analysis of (**F**) *SLC7A11* (*n* = 3), (**G**) *GCLM* (*n* = 3), and (**H**) *GLS* protein expression after the stimulation with 5 µg/mL hLcn-2 for the indicated time points. Nucleolin was analyzed as loading control. A representative picture (upper panels) from 4 independent experiments is given along with the densitometrical analysis (lower panels) (*n* = 3). Graphs are displayed as means ± SEM with * *p* < 0.05, ** *p* < 0.01, *** *p* < 0.001.

Taking other stress responses, besides ROS-formation, into account, we explored activation of the integrated stress response (ISR) upon hLcn-2 stimulation. First, we analyzed the phosphorylation of eIF2α as central ISR component. We noticed enhanced eIF2α phosphorylation already at 1 h after hLcn-2 stimulation, which remained high at 8 h and returned to control levels at 24 h (Figure 4A). In line, expression of activating transcription factor 4 (ATF4), which increases in response to phosphorylation, i.e., inactivation, of eIF2α, was elevated at 1 and 8 h after hLcn-2 stimulation (Figure 4B). The 8 h timepoint coincided with induction of the typical ATF4 target gene *CHOP* (C/EBP homologous protein) at mRNA level (Figure 4C). Taking ATF4 expression in response to hLcn-2 into account, we asked whether there is a direct link to the targets of interest. Therefore, we knocked down *ATF4* (siATF4) in renal CAKI1 tumor cells and compared the expression of *SLC7A11*, *GCLM*, and *GLS* after 1 and 24 h of hLcn-2 stimulation to scRNA-treated cells (Figure 4D). *ATF4* knockdown efficiency was controlled by Western analysis (Supplemental Figure S4A) and ATF4 target gene expression (Supplemental Figure S4B). With respect to *SLC7A11*, *GCLM*, and *GLS* expression, only *SLC7A11* was controlled by ATF4 at 24 h, whereas both *GCLM* and *GLS* remained unaltered compared to scRNA-treated control cells (Figure 4D). Next, we explored whether ATF4 might be crucial for hLcn-2 to protect from ferroptosis. Therefore, we analyzed the viability of ATF4-knockdown cells after hLcn-2 pre-stimulation and a subsequent erastin challenge (Figure 4E). In cells lacking ATF4, hLcn-2 no longer protected from erastin-induced cell death, pointing towards a crucial role of the ISR pathway in mediating the protective effects of hLcn-2 against ferroptosis.

Considering that only *SLC7A11* was transcriptionally controlled by ATF4 as well as siATF4-treated cells lost the hLcn-2-induced protective phenotype, we aimed at clarifying protein levels of *SLC7A11*. *SLC7A11* showed enhanced protein expression after hLcn-2 stimulation at 1, 8, and 24 h of treatment (Figure 4F), whereas the other hLcn-2 target genes *GCLM* (Figure 4G) and *GLS* (Figure 4H) remained unaltered.

### 2.5. The ISR Pathway Is Crucial for hLcn-2 to Protect from Ferroptosis

**Figure 5:**
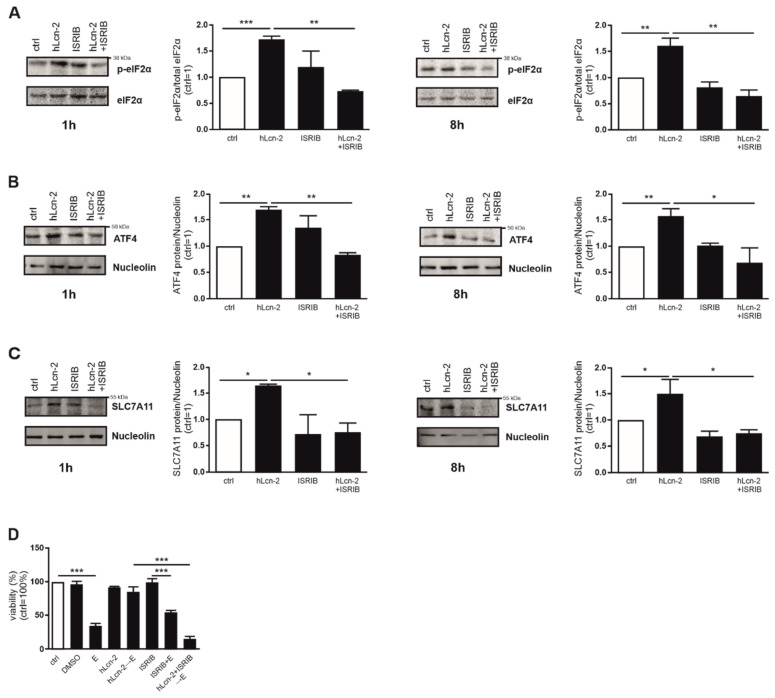
hLcn-2 delays ferroptosis through induction of the integrated stress response. (**A**–**C**) Western analysis of (**A**) p-eIF2α (*n* = 3), (**B**) ATF4 (*n* = 3), and (**C**) *SLC7A11* protein expression after the co-stimulation of hLcn-2 (5 µg/mL) and the inhibitor of the integrated stress response (ISRIB; 1 µM) for the indicated time points. Either total eIF2α or nucleolin was analyzed as loading control. A representative picture (left panels) from 4 independent experiments is given along with the densitometrical analysis (right panels) (*n* = 3). (**D**) Cell viability measured with CellTiter Blue assay, normalized against the unstimulated control. DMSO served as solvent control. Erastin (10 µM) was used to induce ferroptosis for 24 h. CAKI1 cells were co-stimulated with 5 µg/mL hLcn-2 and 1 µM ISRIB for 24 h, washed, and incubated with erastin (10 µM) for additional 24 h (*n* = 4). Graphs are displayed as means ± SEM with * *p* < 0.05, ** *p* < 0.01, *** *p* < 0.001.

In order to explore whether *SLC7A11* expression via the ISR pathway is crucial for hLcn-2 to protect renal tumor cells, we analyzed phosphorylation of eIF-2α in the presence or absence of the integrated stress response inhibitor (ISRIB) after 1 and 8 h of hLcn-2 stimulation (Figure 5A). eIF-2α phosphorylation in response to hLcn-2 was sensitive to ISRIB for all timepoints, whereas ISRIB alone only provoked minor effects. In line, ATF4 expression after hLcn-2 addition was significantly reduced upon co-stimulation of hLcn-2 and ISRIB at 1 and 8 h (Figure 5B). To find out if *SLC7A11* is a direct target of the ISR, we analyzed its protein expression in the presence of ISRIB. Indeed, *SLC7A11* protein expression was significantly reduced by ISRIB at 1 h as well as 8 h after hLcn-2 stimulation (Figure 5C). To prove that the ISR is crucial for hLcn-2 to protect from ferroptosis, we pre-stimulated CAKI1 cells with hLcn-2 in the presence of ISRIB and subsequently induced ferroptotic cell death by erastin. Inhibiting the ISR with ISRIB blocked protection towards ferroptosis seen with hLcn-2 (Figure 5D).

Conclusively, hLcn-2 promotes ROS formation and the subsequent degradation of Keap-1, thus allowing *Nrf2* to activate its target genes *SLC7A11*, *GCLM*, and *GLS*. hLcn-2 also fosters the ISR through eIF-2α phosphorylation and activation of its target ATF4 to control transcription of *SLC7A11*. Furthermore, we showed that only *SLC7A11* is translated and enhanced at protein level upon hLcn-2 stimulation.

### 2.6. Correlative Expression of hLcn-2 and SLC7A11 in Clear Cell RCC Patients

**Figure 6:**
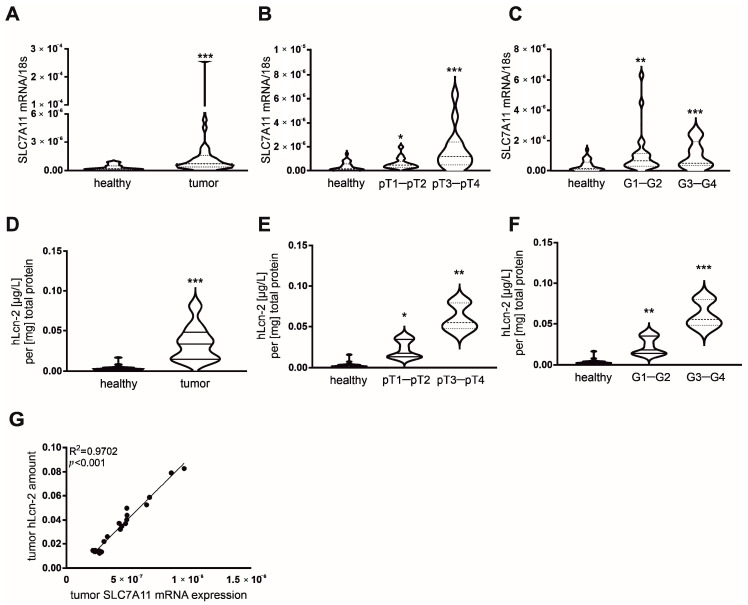
*SLC7A11* mRNA expression correlates with the amount of iron-loaded Lcn-2 in tumor tissue of ccRCC patients. (**A**) mRNA expression of *SLC7A11* in samples of tumor tissue of ccRCC patients (*n* = 32) compared to samples of adjacent healthy tissue of the same patients. (**B**,**C**) *SLC7A11* mRNA expression in healthy tissue, (**B**) G1–G2 (*n* = 23) and G3–G4 (*n* = 9) tumor grade and (**C**) pT1–pT2 (*n* = 18) and pT3–pT4 (*n* = 14) tumor stage. mRNA expression was normalized to housekeeping gene *18S* expression. (**D**) The amount of hLcn-2 was analyzed by AAS measurements in Lcn-2-immunoprecipitated samples of tumor tissue of ccRCC patients (*n* = 25) and compared to samples of adjacent healthy tissue of the same patients. (**E**,**F**) Lcn-2-bound iron in healthy tissue, (**E**) G1–G2 and G3–G4 tumor grade, and (**F**) pT1–pT2 and pT3–pT4 tumor stage (*n* = 25). (**G**) Correlation analysis between *SLC7A11* mRNA expression and the amount of AAS-detected hLcn-2 in tumor tissue of ccRCC patients (*n* = 25). Significance of the correlation was determined by Spearman’s test including all investigated groups. Graphs are displayed as (**A**–**F**) violin plots, indicating the median and quartiles with whiskers reaching up to 1.5 times the interquartile range and (**G**) simple linear regression of correlations, with * *p* < 0.05, ** *p* < 0.01, *** *p* < 0.001.

To further explore our findings in a clinically relevant setting, we analyzed the hLcn-2-dependent target genes *SLC7A11*, *GCLM*, and *GLS* at mRNA level in either tumor tissue or adjacent healthy tissue of 32 patients diagnosed with clear cell RCC (ccRCC). Clinical data of patients are given in Supplemental Figure S5A. We observed enhanced *SLC7A11* mRNA expression in tumor tissue compared to adjacent healthy tissue (Figure 6A), while mRNA of *GCLM* (Supplemental Figure S5B) and *GLS* (Supplemental Figure S5C) remained unaltered. *SLC7A11* expression significantly correlated with both pathological tumor (pT) stage and tumor grade (Figure 6B,C), showing a significant increase in low pT stages (pT1-pT2) as well as low tumor grades (G1–G2) with further enhancement in higher pT stages (pT3-pT4) as well as higher tumor grades (G3–G4) compared to healthy adjacent control tissue from the same patient. We also checked the amount of iron-loaded Lcn-2 in ccRCC tumor tissues compared to adjacent healthy tissues (Figure 6D). To this end, we immunoprecipitated Lcn-2 from patient-derived tissue and subsequently measured its iron-load by atomic absorption spectroscopy (AAS). There were significantly enhanced hLcn-2 levels in tumor compared to adjacent healthy tissues (Figure 6D). Furthermore, we found increased levels of hLcn-2 in low tumor pT stage (pT1–pT2) as well as low tumor grades (G1–G2) and noticed a further increase in higher pT stages (pT3–pT4) as well as higher tumor grades (G3–G4) (Figure 6E,F). In order to link the expression of *SLC7A11* to the presence of hLcn-2 in tumor tissue, we correlated *SLC7A11* mRNA expression to hLcn-2 levels and found a significant association between these two parameters (Figure 6G).

Our results suggest that iron-loaded Lcn-2 enhances the survival of renal tumor cells through ROS formation and the activation of the ISR pathway with a subsequent increased *SLC7A11* expression, which adds to attenuate ferroptotic cell death.

## 3. Discussion

Although the iron-load of Lcn-2 in defining its biological activity was described in a variety of studies [21,31,32], we now provide mechanistic details explaining its pro-tumor characteristics. We identified a cluster of genes of the glutathione biosynthesis as well as antioxidant pathway that are integral to hLcn-2-dependent signaling. We provide evidence that a coordinated response of ROS besides induction of the ISR/p-eIF2α/ATF4-axis fosters protection against erastin-induced ferroptosis.

Ferroptosis is an iron-dependent form of programmed cell death, which relies on ROS, that is triggered by a diverse set of physiological and pathophysiological conditions [11]. For cancer cells, it was speculated that ferroptosis adds to anti-tumoral mechanisms by induction of cell death in nutrient-deficient or stressed cells [25]. Although cancer cells suffer from continuous oxidative stress, and ROS are considered causative for the induction of ferroptosis, ferroptotic cell death is not commonly happening during tumor development. This inability to undergo ferroptotic death might be explained by the ability of cancer cells to cope with catalytic iron and to maintain a fine balance of labile iron, which fosters cell proliferation as well as metabolic adaptation, and thiols that neutralize toxic redox-active iron [33,34]. Since ferroptosis is linked to a dysregulated antioxidant system, reduced GSH and the transcription factor *Nrf2* play critical roles during ferroptosis execution [35,36]. Hence, depletion of GSH causes the generation of lipid ROS in the presence of iron [5]. In turn, many genes of the antioxidant cellular machinery are transcriptional targets of *Nrf2*, including the cystine-glutamate antiporter xCT (*SLC7A11*) and glutathione peroxidase (GPX) 4 [37].

*Nrf2* does not only target genes of the antioxidant pathway to limit ROS-induced stress, but also provides a link to cellular iron homeostasis by regulating a variety of iron-dependent genes, including the Tf-TfR system [38], *HO-1* [39], and the iron exporter ferroportin (FPN) [40]. Thereby, Nrf2 activation decreases the sensitivity of cancer cells to ferroptosis [41]. Along these lines, it was shown that *Nrf2* signaling enhances resistance of head and neck carcinoma cells to artesunate, whereas *Nrf2* inhibition allows for efficient ferroptosis-induction in resistant head and neck cancer [42]. In the present study, we found that iron-loaded Lcn-2 fosters activation of the *Nrf2* pathway, with concomitant induction of the *Nrf2* target genes *SLC7A11*, *GCLM*, and *GLS*, all of which are known to orchestrate the ferroptotic cell death response. It can be speculated that the Lcn-2-transported iron is delivered to cells, which, in turn activates *Nrf2* as one of the central oxidant/antioxidant sensors. However, iron delivered by the Tf-TfR system remained inefficient in activating *Nrf2* targets, although elevating intracellular iron as efficiently as hLcn-2. Moreover, protection from ferroptosis was seen with hLcn-2, but was not recapitulated by hTf or iron-free Lcn-2 (mutant or apo form). Therefore, we conclude that iron per se is not sufficient to trigger the *Nrf2*-dependent adaptive survival response that occurred after hLcn-2 stimulation.

We believe that hLcn-2 adopts a specific conformation in its iron-bound form that either activates specific signaling molecules or interacts with or binds to cellular proteins to modulate their activation. Supporting this hypothesis, Huang et al. recently showed that the biological activity of Lcn-2 depends on a unique conformational distortion mechanism towards a continuous unfolded-folded state of the Lcn-2 protein in its iron-bound form that triggers the accumulation of ROS as an underlying mechanism of its pathology [43]. As a consequence, we co-stimulated cells with hLcn-2 and NAC to prevent initial ROS-induction to underscore the impertinent role of early ROS-induction for the pro-tumor characteristics of hLcn-2. However, we found that erastin-induced ferroptosis could not be rescued by co-stimulation of hLcn-2 with NAC, implying additional stress-associated molecular mechanisms that are hLcn-2 responsive.

Ferroptosis-inducing ROS are detoxified by GSH. As an outstanding cellular antioxidant, most of its cytoprotective effects are attributed to redox homeostasis [44]. Unfortunately, the effect of cysteine limitation towards regulation of ferroptosis is still not fully understood. It might be speculated that cysteine availability adopts a more important role than simply being a critical substrate for GSH synthesis. Our RNAseq data showed enrichment of genes associated with the GO-term: regulation of cysteine metabolic process. In line, hLcn-2 induced expression of *SLC7A11*, a key component of the Xc-antiporter system that exchanges extracellular cystine for intracellular glutamate. Considering the importance of *SLC7A11* for sufficient cysteine supply and GSH biosynthesis, it is not surprising that inhibition of *SLC7A11* depleted GSH, thereby enhancing ferroptosis [45].

Moreover, cysteine deprivation activates the ISR [46], which causes phosphorylation of eIF2α to interfere with general protein synthesis. On the other side, ISR induces translation of selective mRNAs, including ATF4 [47]. One of the target genes of the transcription factor ATF4 is *SLC7A11*, which, in turn, fosters ISR protective effects [47]. ATF4 was described as a negative regulator of ferroptosis in glioma cells [48] and its forced expression in hepatoma cells induced protection from ferroptosis [49]. Our results confirmed that activation of the ISR with subsequent ATF4 induction was implied in promoting resistance of renal tumor cells against ferroptotic cell death. Furthermore, we applied ISRIB, an ISR inhibitor, to prove the pivotal role of the ISR for hLcn-2-mediated protection. Our data imply that *SLC7A11* is one of the targets that is selectively translated after global translation inhibition through phosphorylation of eIF-2α. Indeed, it was previously described that the ISR response, with concomitant activation of ATF4 and its targets were associated with poor prognosis and therapy resistance in cisplatin-resistant gastric cancer [50], temozolamide chemoresistance in human gliomas [51], as well as paclitaxel-resistant breast cancer [52]. Likely, ISR-mediated up-regulation of *SLC7A11* might be considered as molecular executer of malignancy and therapy resistance in response to hLcn-2. To corroborate this hypothesis, we observed a significant correlation between *SLC7A11* expression and the amount of iron-loaded Lcn-2 in tumor tissue of ccRCC patients. Still, we cannot exclude other stress-induced signaling pathways to be involved in Lcn-2-mediated protection against ferroptosis. Interestingly, the antioxidant activity of paraoxonases might adopt a pivotal role for the development of chemo- and radiation-therapy, especially in renal cancer. Paraoxinase 1 (PON1) was recently described to be involved in the development of sunitinib resistance in renal cancer [53,54]. Of note, it was already described that paraoxonases also prevent iron-ascorbate-induced ROS [55].

Moreover, Liu et al. found that the stress-responsive transcription factor nuclear protein 1 (NUPR1) transactivates Lcn-2 in pancreatic cancer cells, which, in turn, induces ferroptosis resistance in vitro and in vivo [56]. Therefore, the role of endogenous, cancer cell-expressed Lcn-2 might be detrimental for ferroptosis susceptibility. However, no details about the iron-load of cancer cell-expressed Lcn-2 are available at the moment. We hypothesize that cancer cells secrete Lcn-2 in its iron-free form, which might be rapidly taken up to diminish stress-induced cellular death. On the other hand, iron-loaded Lcn-2, which comes from other sources within the tumor microenvironment, adds to the protective effects by inducing antioxidant pathways. Along these lines, we recently showed that renal tubular cells secrete iron-free Lcn-2 upon sepsis-induced damage, whereby Lcn-2 serves as a marker of renal injury [57]. In contrast, iron-loaded Lcn-2 was mainly secreted by renal macrophages, serving as a marker for renal regeneration. Further studies that take the role of the Lcn-2 iron-load into account are urgently needed to better understand the diverse functions of Lcn-2.

Our study underscores the significance of the iron-load in defining the biological activity of Lcn-2. We provide evidence that iron-loaded Lcn-2 not only induces the ROS-*Nfr2* axis, but also fosters ISR-dependent expression of *SLC7A11* to attenuate erastin-induced ferroptosis in renal cancer.

## 4. Materials and Methods

### 4.1. Cell Culture

Human renal *CAKI1* and A498 as well as mammary *MCF*-7 tumor cells were cultured in Dulbecco’s modified Eagle medium (DMEM) with high glucose (Gibco, Dreieich, Germany), supplemented with 100 U/mL penicillin (Sigma-Aldrich, Taufkirchen, Germany), 100 µg/mL streptomycin (Sigma-Aldrich), and 10% fetal bovine serum (FBS; Capricorn Scientific, Ebersdorfergrund, Germany). Tumor cells were cultivated in a humidified atmosphere with 5% CO_2_ at 37 °C and passaged 3 times per week. 24 h prior to stimulation, cells were serum starved. Cells were routinely tested for mycoplasma.

Human renal tubular epithelial cells (TEC) as well as renal tumor tubular epithelial cells (TTEC) were isolated as previously described [58,59]. Briefly, tumor tissue was minced and digested with collagenase/dispase (1 mg/mL) for 45 min at 37 °C. Digested fragments were passed through a 106-µm mesh and incubated with collagenase (1 mg/mL), DNase (0.1 mg/mL) and MgCl_2_ (5 mmol/L). The cell pellet was washed with Hank’s buffered salt solution after Percoll density-gradient centrifugation. Cells were seeded on FCS-precoated plates and grown in M199 medium (Sigma-Aldrich) supplemented with penicillin (100 U/mL), streptomycin (100 mg/mL) and 10% FCS. TTEC medium was changed every 3 days, and cells were passaged when confluence was reached. Passages between 2 and 4 were used for experiments.

### 4.2. Treatments

Cells were stimulated with 5 µg/mL Lcn-2, either in its iron-free apo-form, the iron-loaded holo-form, or a no iron-binding mutant-form, for 24 h. As controls, we included the iron-transporting protein holo-transferrin (hTf, 5 µg/mL, Sigma-Aldrich) as well as iron (III) chloride (FeCl_3_; 25 µM, Sigma-Aldrich) and Lcn-2-binding pyrocatechol-FeCl_3_-complex (25 µM, Sigma-Aldrich,) to test target gene expression at mRNA level as well as intracellular iron levels by atomic absorption spectroscopy (AAS).

We used both erastin (1–10 µM as indicated in Supplemental Figure S2, Cayman Chemical, Hamburg, Germany) to induce ferroptosis in *CAKI1* cells. We applied ferrostatin-1 (fer-1; 5 µM, Cayman Chemical, Hamburg, Germany), as well as the pan caspase inhibitor z-vad-fmk (carbobenzoxy-valyl-alanyl-aspartyl-[O-methyl]-fluoromethylketone; 10 µM, Cayman Chemical, Hamburg, Germany) as controls as shown in Supplemental Figure S2.

To test the effect of Lcn-2 in preventing ferroptotic cell death, we pre-stimulated *CAKI1* cells with 5 µg/mL Lcn-2 for 24 h and added 10 µM erastin for additional 24 to 72 h for measurements of vitality applying CellTiter Blue and survival by xCELLigence. As controls, we applied DMSO. To control the effect of ROS-induction regarding the cytoprotective effects of hLcn-2 against ferroptosis, we used hydrogen peroxide (H_2_O_2_; 500 µM, AppliChem GmbH, Darmstadt, Germany) or *N*-acetyl-l-cysteine (NAC; 10 mM, Thermo Fisher Scientific, Dreieich, Germany). For the analysis of the implication of the integrated stress response, we applied the inhibitor of integrated stress response (ISRIB; 1 µM, Sigma-Aldrich).

### 4.3. RNA Extraction and Quantitative Real-Time PCR (qRT-PCR)

RNA isolation, cDNA synthesis, and qPCR were performed as previously described [21]. Briefly, the RNeasy Micro Kit (Qiagen, Hilden, Germany) was used for the isolation of RNA. Transcription into cDNA was done with the Sensiscript RT kit (Qiagen). 18S mRNA expression was chosen as an internal housekeeping gene control. Primers were acquired from Biorad (LCN-2, qHsaCED0045408) or from Biomers (Ulm, Germany):

18S rRNA: 5′→3′ GTA ACC CGT TGA ACC CCA, 3′→5′ CCA TCC AAT CGG TAG TAG CG; *SLC7A11* (solute carrier family 7 member 11): 5′→3′ CCT CTA TTC GGA CCC ATT TAG T, 3′→5′ CTG GGT TTC TTG TCC CAT ATA A; *GCLM* (glutamate-cysteine ligase modifier subunit): 5′→3′ GCT GTA TCA GTG GGC ACA G, 3′→5′ CGC TTG AAT GTC AGG AAT GC; *GLS* (glutaminase): 5′→3′ TTC CAG AAG GCA CAG ACA TGG TTG, 3′→5′ GCC AGT GTC GCA GCC ATC AC; ATF4 (activating transcription factor 4): 5′→3′ GTT GCC AAA CAT CCC C, 3′→5′ CGA GGC CTT CAA GGA GG; CHOP (C/EBP homologous protein): 5′→3′ TGG AAG CCT GGA GGA C, 3′→5′ AGG TGC TTG TTC TGC T.

### 4.4. RNA Sequencing

*CAKI1* cells were stimulated with 5 µg/mL holo-Lcn-2 for 24 h. Afterwards, RNA was extracted using the RNeasy Midi Kit (Qiagen). RNA quality was evaluated using an Agilent 2100 Bioanalyzer applying a RNA 6000 Nano Chip (Agilent Technologies, Santa Clara, CA, USA), followed by quantification with a Qubit HS RNA Assay Kit (Thermo Fisher Scientific, Dreieich, Germany). For library preparation, 500 pg of RNA was applied using the QuantSeq 3′ mRNA-Seq Library Prep Kit with the UMI Second Strand Synthesis Module for QuantSeq (Lexogen GmbH, Vienna, Austria). Qubit ds DNA HS Assay Kit (Thermo Fisher Scientific) and Agilent DNA High Sensitivity DNA Chip (Agilent Technologies, Santa Clara, CA, USA) were used to evaluate quantity and quality of the complementary DNA (cDNA) libraries, respectively. Libraries were sequenced (single-end, 75 cycles) using a High Output Kit v2 on a NextSeq 500 sequencer (Illumina, San Diego, CA, USA). Processed reads were aligned to the GRCh38 reference genome build [60] with STAR [61], counted using HTSeq [62], and the differential gene expression analysis was done with edgeR [63] in R 3.4.4 (https://www.r-project.org/ accessed on 14 December 2018), according to the edgeR manual. Annotated DEG were visualized in a heatmap using Morpheus (https://software.broadinstitute.org/morpheus accessed on 5 April 2020). Enriched GO-terms were determined using GOrilla (http://cbl-gorilla.cs.technion.ac.il/ accessed on 13 April 2020). Details of the bioinformatics analyses are provided in the Suppl. Methods. Sequencing data were deposited and made available under the GEO accession number GSE171939.

### 4.5. Western Blot Analysis

Western blot analysis was performed as previously described [64]. Briefly, cells were resuspended in lysis buffer containing 6.65 M urea, 10% glycerol, 1% SDS, and 10 mM Tris-HCl (pH 7.4). The protein content was determined by the Lowry method according to manufacturer’s instructions (Bio-Rad, Munich, Germany) after sonification and centrifugation (15,000× *g*, 5 min). 100 μg protein was boiled in loading buffer, supplemented with 20% glycerol and bromophenolblue, loaded on a SDS gel, and blotted using Immobilion-FL polyvinylidene difluoride (PVDF) membranes (Merck Milli-pore, Schwalbach, Germany). The membranes were blocked with TBST containing 5% milk powder or 5% bovine serum albumin (BSA) and analyzed using a specific antibody against *SLC7A11* (Abcam, Cambridge, UK), *GCLM* (Abcam), *GLS* (Abcam; recognizes endogenous levels of both the 73 kDa KGA and the 65 kDa GAC isoforms), Keap1 (Cell Signaling Technology, MA, USA), p-eIF2α (Cell Signaling Technology), eIF2α (Cell Signaling Technology), GPX4 (Cell Signaling Technology, according to the manufacturer two bands are detected), and ATF4 (Cell Signaling Technology). An antibody against nucleolin (SantaCruz, Heidelberg, Germany) was used as loading control. For densitometrical quantification, the fluorescence intensity was normalized to nucleolin and is given relative to unstimulated controls. All bands were visualized using the Odyssey infrared imaging system (Li-COR Biosciences GmbH, Bad Homburg, Germany).

### 4.6. Viability Assay

Viability was measured with CellTiter Blue (Promega, Walldorf, Germany) following the manufacturer’s instructions (Promega Corporation, Madison, WI, USA). Fluorescence was measured on an Infinite 200 pro plate reader (Tecan, Männedorf, Switzerland).

### 4.7. Glutathione Measurement

Glutathione was measured applying the Glutathione Colorimetric Detection Kit following the manufacturer’s instructions (Thermo Fisher Scientific).

Additionally, reduced glutathione was determined by HPLC analysis after derivatization using Ellman’s reagent (5,5′-dithiobis-(2-nitrobenzoic acid), DTNB), as previously described [65,66]. The method is based on the reaction of the thiol with DTNB to form a mixed disulfide nitro-5-thiobenzoic acid (NTB). Briefly, *CAKI1* cells were stimulated with 5 µg/mL hLcn-2 for 4, 8, and 24h. For detection of intracellular GSH, cells were lysed in 30% (*v*/*v*) acetonitrile/water containing 1 mM DTNB for 20 min and stored at −80 °C until measurement. A total of 50 µL of these samples were subjected to HPLC (high performance liquid chromatography) analysis. Quantification was performed using standards from incubations of DTNB (2 mM) with GSH (10 or 100 µM).

### 4.8. Small Interfering RNA (siRNA) Transfection

To knockdown either *Nrf2* (L-003755-00-0005, Horizon Discovery, Waterbeach, UK) or ATF4 (L-005125-00-0005, Horizon Discovery) in *CAKI1* cells, we used specific siRNA clones and the hyperfect transfection reagent (Qiagen) following the manufacturer’s instructions. A non-targeting, scrambled siRNA (sictrl; D-001810-10-20, Horizon Discovery, Waterbeach, UK) was applied as control.

### 4.9. Atomic Absorption Spectrometry (AAS)

Iron measurements were performed as previously described [57]. Cells were harvested in lysis buffer (62.5 mM Tris-HCl pH 6.8, 50% glycerol, 2% SDS). The iron amount was quantified relative to the total protein content.

### 4.10. ROS Measurements

A LSRII/Fortessa flow cytometer (BD, Heidelberg, Germany) was used to quantify ROS production. The fluorescent dye 2′,7′-dichlorodihydrofluoresceindiacetate (H2DCF-DA; 10µM, Molecular Probes, Eugene, OR, USA) was used. Samples were expressed as mean fluorescence intensity (MFI) and fluorescence minus one (FMO) controls were used.

### 4.11. Generation of Recombinant Lcn-2 and Complex Formation

Generation of recombinant Lcn-2 and complex formation were performed as previously described [31]. Transformation of *E. coli* (New England Biolabs, Frankfurt am Main, Germany) with either a pGEX-4T3-Lcn-2 or the pGEX-4T3_LCN-2_mutant plasmid, both containing an insert for human Lcn-2 tagged with glutathione-S-transferase (GST) for purification, was performed in order to produce recombinant human Lcn-2. Initiation of Lcn-2 expression in bacterial cultures was achieved by supplementing isopropyl-β-D-thiogalactopyranosid (IPTG, Sigma-Aldrich). For purification of Lcn-2 Pierce Glutathione Agarose (Thermo Fisher) was used. To eliminate bacterial endotoxins Detoxi-Gel Endotoxin Removing Resin (Thermo Fisher) was utilized.

The Lcn-2 mutant (mLcn-2) with no iron-binding capacity was generated as described before [31]. Briefly, point mutations were inserted into pGEX-4T3_Lcn-2 plasmids. The mutated plasmids were used for transformation of *E. coli* (New England Biolabs, Frankfurt, Germany) to produce recombinant human mLcn-2.

Complex formation of Lcn-2 and iron-catechol was monitored by UV-vis-spectrometry in the range of 300 to 800 nm. The iron-free (apo-) protein spectrum was obtained from a 6.25 µM Lcn-2 solution. For the generation of iron-loaded (holo-) Lcn-2 and mLcn-2, the protein solution was mixed with an aliquot of 5 mM iron-catechol (3:1 mol:mol) solution, resulting in equimolar concentration of Lcn-2 and iron-catechol.

### 4.12. Lcn-2 Immunoprecipitation (IP)

Immunoprecipitation was performed according to the protocol previously described by Rehwald et al. [31]. Briefly, human tumor tissue samples were snap frozen, homogenized, and sonicated. After centrifugation, the supernatant was immunoprecipitated by applying an Lcn-2 specific antibody (R&D systems, Wiesbaden, Germany) and bound to magnetic beads (Thermo Fisher). After elution, the iron amount was analyzed by AAS.

### 4.13. Survival Assays

Survival assays were performed using the xCELLigence RTCA DP instrument (OLS, Bremen, Germany) as previously described [27]. Survival was recorded continuously for 3 days and the relative slope was calculated over time. Data were acquired as a measure for time-dependent impedance changes. RTCA Software 1.2 (OLS) was used for acquisition and analysis.

### 4.14. Ethics

Studies were conducted according to the ethical standards of the Declaration of Helsinki as well as national and international guidelines. The local Ethics Committee of the Goethe—University Hospital Frankfurt am Main (file number 04/09 UGO 03/10) and Philipps—University Hospital Marburg (file number 122/14) approved both study protocol and consent documents. All consent documents were filed in a centralized manner by the university cancer center (UCT) Frankfurt.

### 4.15. Participants

Whenever possible, tumor and corresponding adjacent healthy tissues were obtained from patients with histopathological diagnosis of renal cancer (see Supplemental Figure S5A). Via imaging by CT and/or MRI preoperative staging was accomplished for all patients. Surgery was performed before receiving other therapy. All tumor and adjacent healthy renal tissues were collected between 2016 and 2019 immediately after nephrectomy. So far, no follow-up data were available. Samples were aliquoted and either immediately processed for single cell suspensions, fixed in 4% paraformaldehyde (PFA) for immunohistological analysis, or stored at −80 °C until further processing. For pathological examination, the current UICC TNM classification of malignant tumors was applied.

### 4.16. Statistical Analysis

Prism software (GraphPad Inc., San Diego, CA, USA, version 8.2.1) was used to perform statistical analysis. Statistically significant differences were calculated applying either Mann-Whitney test for two sample comparison or Kruskal–Wallis tests followed by Dunn’s correction for multiple comparisons. Data are expressed as means ± SEM or violin plots, indicating the median and quartiles with whiskers reaching up to 1.5 times the interquartile range. *p* values were considered significant at * *p* < 0.05, ** *p* < 0.01, *** *p* < 0.001.

## Data Availability

Sequencing data were deposited and made available under the GEO accession number GSE171939.

## Supplementary Materials

The following are available online at https://www.mdpi.com/article/10.3390/metabo11050329/s1, Figure S1: hLcn-2 induces *SLC7A11*, *GCLM*, and *GLS* in renal A498 and mammary MCF-7 breast cancer cells as well as comparison of primary human tubular epithelial cells (TEC) to primary renal tumor cells (TTEC), Figure S2: CAKI1 tumor cells are sensitive to erastin-induced cell death and GPX4 is induced after hLcn-2 stimulation, Figure S3: Nrf2 knockdown efficiency after hLcn-2 stimulation, Figure S4: ATF4 knockdown efficiency after hLcn-2 stimulation, Figure S5: *GCLM* and *GLS* mRNA expression in ccRCC patients, Extended supplemental methods: RNA sequencing.
