# Supplementary material for: Iron-Bound Lipocalin-2 Protects Renal Cell Carcinoma from Ferroptosis
Source: Metabolites. 2021 May 19;11(5):329. doi: 10.3390/metabo11050329 (PMC8161288; doi:10.3390/metabo11050329)
Supplement: Supplementary file 1 [file metabolites-11-00329-s001.zip › metabolites-1194385-supplementary.pdf]

metabolites

MDPI

Supplementary Material

Iron-Bound Lipocalin-2 Protects Renal Cell Carcinoma from Ferroptosis

Julia K. Meier [1], Matthias Schnetz [1], Susanne Beck [2], Tobias Schmid [1], Monica Dominguez [1], Sanela Kalinovic [3], Andreas Daiber [3], Bernhard Brüne [1,4,5,6], Michaela Jung [1*]

A

Gene	logFC	p-value	FDR
RHOU	-1.65	9.79E-06	0,023
AUTS2	-1.52	1.95E-06	0,010
C1QBP	-1.02	0.038001	0,038
CAVIN2	-0.85	6.23E-09	0,003
TXNIP	-0.58	1.91E-05	0,023
RRM2	-0.56	2.55E-05	0,011
SNAPC1	-0.53	3.31E-07	0,021
ARHGAP29	-0.49	9.85E-08	0,011
LINCO1111	-0.49	4.98E-06	0,011
COPS9	-0.44	2.93E-05	0,044
GCLM	0.41	1.07E-05	0,011
GLS	0.43	0.038001	0,038
SLFN5	0.47	3.34E-05	0,039
SERPINH1	0.47	1.03E-06	0,010
FADS2	0.49	1.05E-07	0,003
AFF1	0.51	1.94E-05	0,026
PSAT1	0.51	2.08E-05	0,010
DDX41	0.53	0.000167	0,026
HES1	0.54	6.70E-06	0,010
SNX18	0.55	1.99E-05	0,021
GPT2	0.56	7.44E-06	0,004
MRE11	0.58	0.038755	0,039
SCD	0.58	1.65E-05	0,017
CEBPB	0.58	7.53E-05	0,044
SCARNA22	0.59	5.84E-05	0,008
TSEN15	0.61	4.38E-07	0,023
SESN2	0.62	2.30E-06	0,003
SHMT2	0.65	3.70E-11	0,000
FMC1	0.68	4.70E-07	0,021
VASN	0.68	8.34E-07	0,011
SNTB1	0.68	6.25E-06	0,013
CHAC1	0.69	3.13E-06	0,027
MTHFD2	0.70	2.21E-05	0,021
SLC7A11	0.73	2.63E-08	0,008
DNMT3A	0.77	0.000304	0,027
CTHRC1	0.88	7.29E-11	0,003
INHBE	1.04	3.16E-06	0,010
IL21R	1.07	9.18E-08	0,023

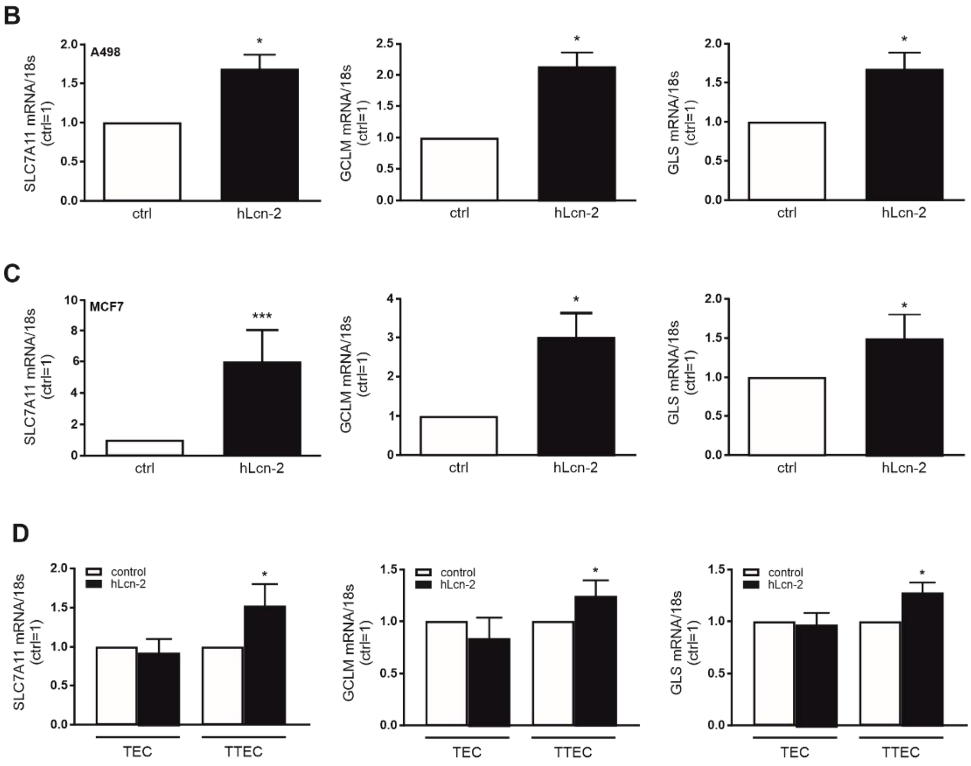

Figure S1. hLcn-2 induces *SLC7A11*, *GCLM*, and *GLS* is only induced in tumor cells. (**A**) Table of all differentially expressed genes after hLcn-2 stimulation showing log2 fold change (logFC) and respective p-value as well as the Benjamini-Hochberg adjusted p-value (FDR). (**B**–**C**) mRNA expression of *SLC7A11* (left), *GCLM* (middle), and *GLS* (right) after 24 h of hLcn-2 (5 µg/mL) in (**B**) renal

A498 tumor cells ($n = 4$) and (**C**) mammary MCF-7 cancer cells ($n = 4$). (**D**) Comparison of primary human tubular epithelia cells (TEC) to primary renal tumor cells (TTEC). mRNA expression was normalized to housekeeping gene 18S expression. Graphs are displayed as means ± SEM with $^{*}p < 0.05$, $^{***}p < 0.001$.

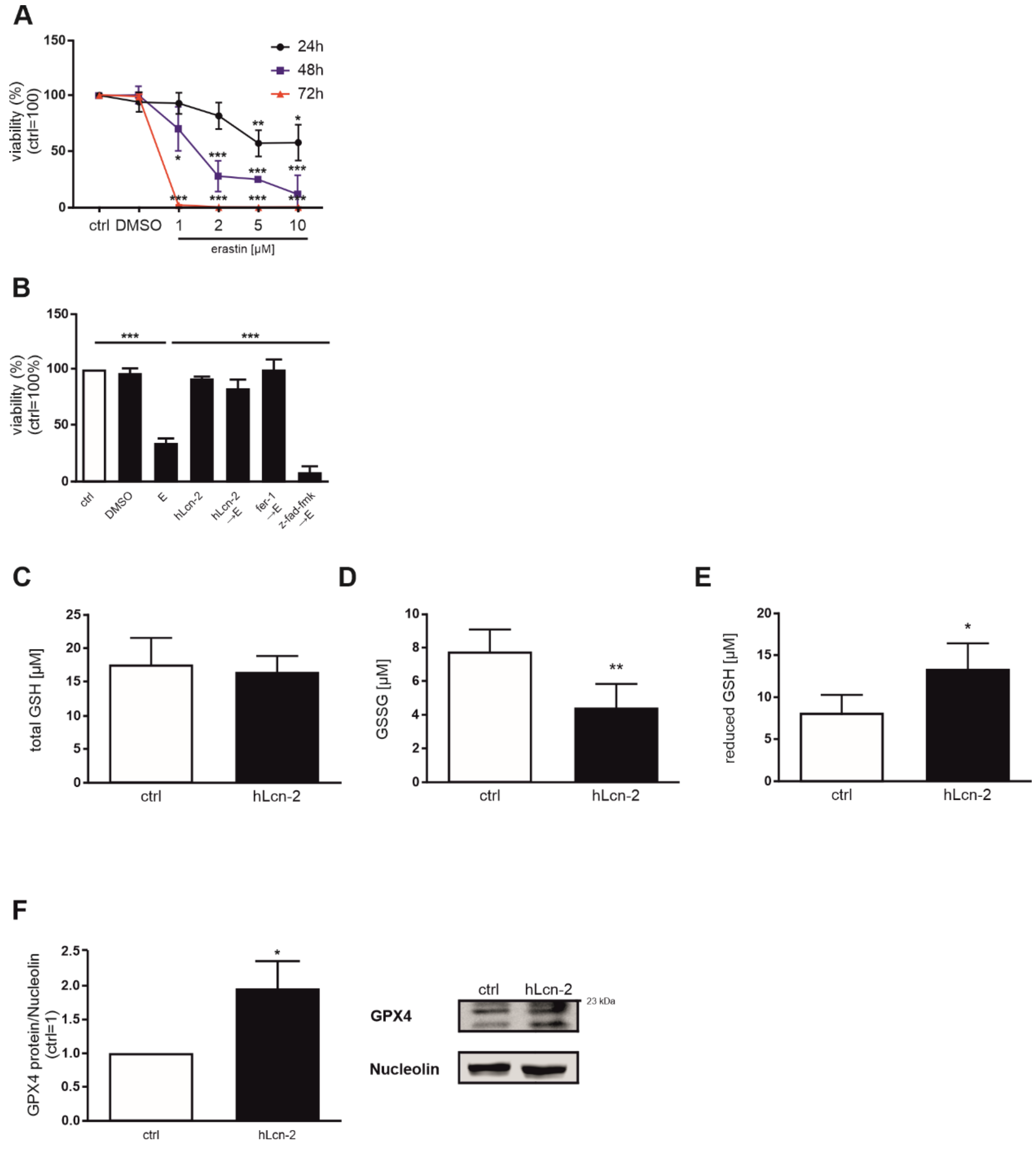

Figure S2. *CAKI1* tumor cells are sensitive to erastin-induced cell death. (**A**–**B**) Cell viability measured with CellTiter Blue assay and normalized to the unstimulated control. DMSO served as solvent control. (**A**) Erastin (1, 2, 5, or 10 µM) was used to induce ferroptosis for 24 h, 48 h, and 72 h ($n = 4$). (**B**) *CAKI1* cells were pre-stimulated with either 5 µg/mL hLcn-2, 5 µM ferrostatin-1 (fer-1), or 10 µM z-vad-fmk for 24 h, washed and incubated with erastin (10 µM) for additional 24 h ($n = 4$). (**C**–**E**) Analysis of (**C**) total GSH ($n = 4$), (**D**) GSSG ($n = 4$), and (**E**) reduced GSH ($n = 4$) by colorimetric detection in *CAKI1* cells stimulated with 5 µg/mL hLcn-2 for 24 h. (**F**) Western blot analysis of *GPX4* in *CAKI1* cells stimulated with 5 µg/mL hLcn-2 for 24 h. Nucleolin was applied as loading control ($n = 4$). Graphs are displayed as means ± SEM with $^{*}p < 0.05$, $^{**}p < 0.01$, $^{***}p < 0$.

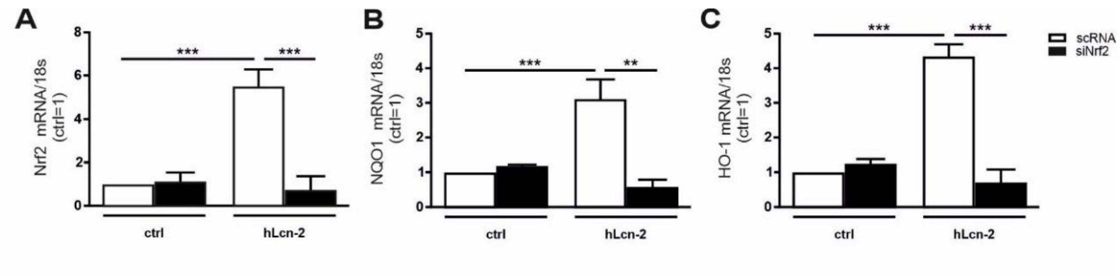

Figure S3. *Nrf2* knockdown efficiency after hLcn-2 stimulation. (**A**–**C**) CAKI1 cells were treated with either a siRNA to knockdown Nrf2 (siNrf2) or a scrambled control RNA (scRNA). mRNA expression of (**A**) *Nrf2* (n = 4), (**B**) NQO1 (n = 4), and (**C**) HO-1 (n = 4) was measured after 24 h of hLcn-2 (5 µg/mL). mRNA expression was normalized to housekeeping gene 18S expression. Graphs are displayed as means ± SEM with $^{**}p < 0.01$, $^{***}p < 0.001$.

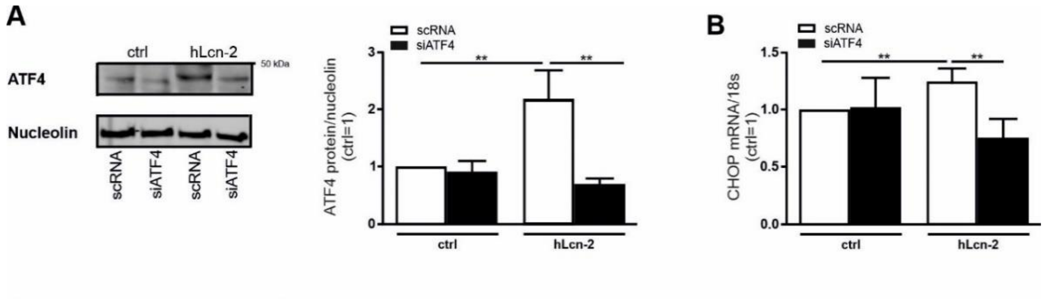

Figure S4. *ATF4* knockdown efficiency after hLcn-2 stimulation. (**A**–**B**) CAKI1 cells were treated with either a siRNA to knockdown *Nrf2* (siNrf2) or a scrambled control RNA (scRNA). (**A**) Western blot analysis of ATF4 protein expression after the stimulation with 5 µg/mL hLcn-2 for 8 h. Nucleolin was analyzed as loading control. A representative picture (left) from at least 4 independent experiments is given along with the densitometrical analysis (right) (n = 3). (**B**) mRNA expression of CHOP after hLcn-2 (5 µg/mL) for 24 h (n = 4). mRNA expression was normalized to housekeeping gene 18S expression. Graphs are displayed as means ± SEM with $^{**}p < 0.01$.

A

	ccRCC
**number of patients**	32
**age (years)**	
mean	64±10
median	65±10
range	42-85
**sex**	
female	22%
male	78%
**pT-stage**	
pT1-pT2	53%
pT3-pT4	47%
**grade**	
G1-G2	82%
G3-G4	18%

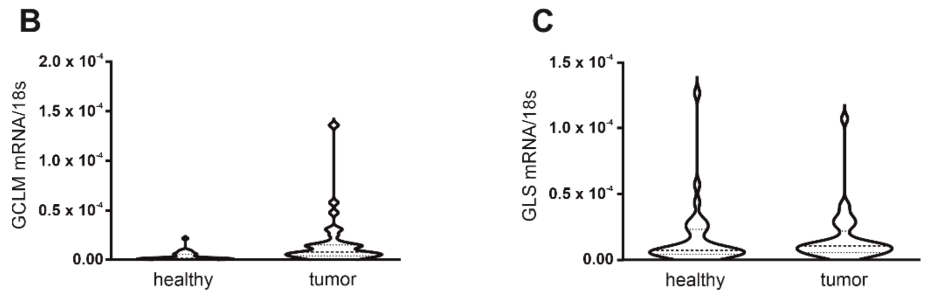

Figure S5: *GCLM* and *GLS* mRNA expression in ccRCC patients

(**A**) Clinical parameters of included patients. (**B**-**C**) mRNA expression of (**B**) GCLM and (**C**) GLS in samples of tumor tissue of ccRCC patients (n=32) compared to samples of adjacent healthy tissue of the same patients. mRNA expression was normalized to housekeeping gene 18S expression. Graphs are displayed as violin plots, indicating the median and quartiles with whiskers reaching up to 1.5 times the interquartile range.

Supplemental Methods

RNA Sequencing-Bioinformatics Analysis

CAKI1 cells were stimulated with 5 µg/ml holo-Lcn-2 for 24 h. Afterwards, RNA was extracted using the RNeasy Midi Kit (Qiagen). RNA quality was evaluated using an Agilent 2100 Bioanalyzer applying a RNA 6000 Nano Chip (Agilent Technologies, Santa Clara, USA), followed by quantification with a Qubit HS RNA Assay Kit (Thermo Fisher Scientific, Dreieich, Germany). For library preparation, 500 pg of RNA was applied using the QuantSeq 3' mRNA-Seq Library Prep Kit with the UMI Second Strand Synthesis Module for QuantSeq (Lexogen GmbH, Vienna, Austria). Both Qubit ds DNA HS Assay Kit (Thermo Fisher Scientific) and Agilent DNA High Sensitivity DNA Chip (Agilent Technologies) were used to evaluate quantity and quality of the complementary DNA (cDNA) libraries. Using the High Output Kit v2 on a NextSeq 500 sequencer (Illumina, San Diego, USA), the libraries were sequenced (single-end, 75 cycles) using a High Output Kit v2 on a NextSeq 500 sequencer (Illumina, San Diego, USA). First FASTQ files were merged to the respective sample. Then, the UMI identifier were attached as index to the read sequence identifier line in the FASTQ file with the umi2index tool. Indexed fastq files were aligned with STAR to the GRCh38 reference genome build. Afterwards, reads in BAM files were collapsed by UMI identifier in the read name with the collapse_UMI_bam tool. Raw read counts were generated with HTSeq. Counts were merged and genes with zero counts in all samples were removed. Counts were filtered to keep only those genes that have at least 1 read per million in at least 2 samples. Differential gene expression analysis was done with edgeR in R 3.4.4 (https://www.r-project.org/, accessed on December, 14th 2018), according to the edgeR manual. Lists of differentially expressed genes (DEG) were annotated with R Bioconductor human annotation data package org.Hs.eg.db. The annotated DEG were visualized in a heatmap using Morpheus (https://software.broadinstitute.org/morpheus, accessed on April, 5th 2020).

A table of highly-enriched gene ontology (GO)-terms was created. Enrichment is defined as: (b/n)/(B/N) (N = total number of genes, B = total number of genes associated with a specific GO-term, n = number of genes in the top of the target set, b = number of genes in the intersection).
